# Supplementary material for: Role of Hyaluronan in Human Adipogenesis: Evidence from in-Vitro and in-Vivo Studies
Source: Int J Mol Sci. 2019 May 31;20(11):2675. doi: 10.3390/ijms20112675 (PMC6600677; doi:10.3390/ijms20112675)
Supplement: Supplementary file 1 [file ijms-20-02675-s001.pdf]

Table S1. Characteristics of the patients chosen for the clinical study [20]. Values are given as the mean ± standard deviation. Comparisons between groups was undertaken by ANOVA. *p*-value < 0.05 significant; ns, not significant.

Characteristic	Healthy (*n* = 20)	Overweight (*n* = 20)	Obese (*n* = 20)	*p*-value
BMI (kg/m^2)	22.39 ± 1.52	27.38 ± 1.28	33.92 ± 2.13	0.0001
Age (Years)	30.95 ± 2.89	30.85 ± 3.44	30 ± 3.29	ns
Free T4 (pmol/L)	13.45 ± 1.28	13.73 ± 1.96	13.7 ± 1.13	ns
TSH (mIU/L)	1.61 ± 0.64	1.39 ± 0.54	1.78 ± 0.71	ns
Free T3 (pmol/L)	4.28 ± 0.4	4.06 ± 0.5	4.38 ± 0.31	ns
Cholesterol (mmol/L)	4.78 ± 0.61	4.79 ± 0.88	5.06 ± 0.62	ns
Triglyceride (mmol/L)	0.74 ± 0.23	0.87 ± 0.28	1.24 ± 0.42	0.0001
High-Density Lipoprotein (mmol/L)	1.46 ± 0.27	1.25 ± 0.3	1.19 ± 0.28	0.01
Insulin (μU/L)	5.26 ± 1.41	6.35 ± 2.98	7.43 ± 2.23	0.01

Table S2. Primer used for this study.

Gene	Forward Primer	Reverse Primer
hAPRT	GCTGCGTGCTCATCCGAAAG	CTTTAAGCGAGGTCAGCTGC
hLPL	GAGATTTCTCTGTATGGACC	CTGCAAATGAGACACTTTCTC
hPPARγ	CAGTGGGGATGTCTCATAA	CTTTTGGCATACTCTGTGAT

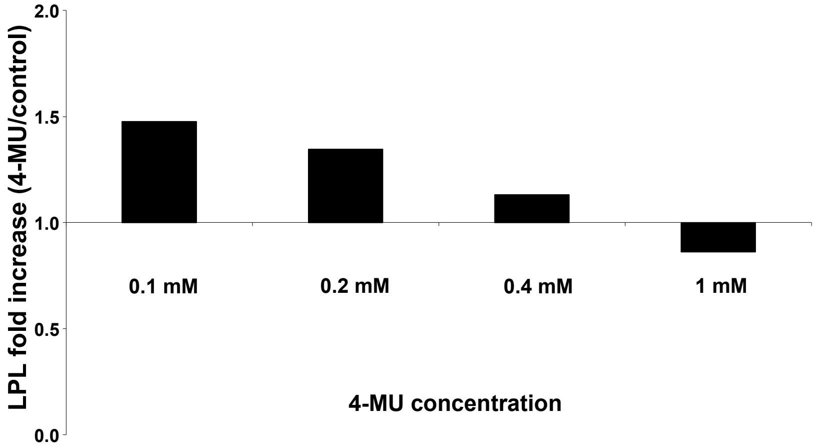

Figure S1. Effect of different concentrations of 4-MU on the adipogenesis of primary subcutaneous PFs. Confluent primary subcutaneous PFs were cultured in adipogenic medium (ADM) with/without 4-MU for 22 days. Total RNA was prepared, and LPL transcripts (terminal differentiation marker) were measured by Q-PCR expressed as transcript copy number (TCN) per 1000 copies of housekeeper gene (adenosine phosphoribosyl transferase APRT). Changes in LPL expression are expressed as fold increases in comparison to the control.
